# Multistakeholder Needs Assessment to Inform the Development of an mHealth-Based Ultrasound-Guided Breast Biopsy Training Program in Nigeria

**DOI:** 10.1200/GO.20.00353

**Published:** 2020-11-20

**Authors:** Kathleen A. Lynch, Adeleye D. Omisore, Thomas M. Atkinson, Olusola C. Famurewa, Jacqueline A. Vera, T. Peter Kingham, Olusegun I. Alatise, Hedvig Hricak, Elizabeth A. Morris, Elizabeth J. Sutton, on behalf of the, African Research Group for Oncology Collaborative

**Affiliations:** ^1^ Department of Psychiatry and Behavioral Sciences, Memorial Sloan Kettering Cancer Center, New York, NY; ^2^ Department of Radiology, Obafemi Awolowo University and Obafemi Awolowo University Teaching Hospitals Complex, Ile-Ife, Nigeria; ^3^ Department of Surgery, Memorial Sloan Kettering Cancer Center, New York, NY; ^4^ Department of Surgery, Obafemi Awolowo University and Obafemi Awolowo University Teaching Hospitals Complex, Ile-Ife, Nigeria; ^5^ Department of Radiology, Memorial Sloan Kettering Cancer Center, New York, NY

## Abstract

**PURPOSE:**

The incidence of breast cancer is rising in Nigeria, and one major barrier to care is the lack of affordable and appropriate breast cancer diagnosis by ultrasound (US)–guided biopsy. The prohibitive cost of US devices limits their availability in low- and middle-income countries. The emergence of mobile health (mHealth) imaging devices may offer an acceptable low-cost alternative. The purpose of this research was to perform a comprehensive needs assessment to understand knowledge, use, training needs, and attitudes as regards image-guided biopsy in Nigeria to inform the development of an mHealth-based US-guided biopsy training program.

**METHODS:**

A multistakeholder needs assessment was conducted at the Sixth Annual African Research Group for Oncology Symposium. Voluntary anonymous surveys were administered to all attendees. A subset of attendees (ie, surgeons, radiologists, pathologists, and nurses) participated in six focus groups. Survey items and interview guides were developed collaboratively with local and international input.

**RESULTS:**

Surveys focusing on use, training needs, and attitudes regarding US-guided biopsies were completed with a 55% response rate (n = 54 of 98) among participants from 22 hospitals across Nigeria. Respondents expressed dissatisfaction with the way breast biopsies were currently performed at their hospitals and high interest in having their institution participate in a US-guided biopsy training program. Focus group participants (n = 37) identified challenges to performing US-guided procedures, including equipment functionality and cost, staff training, and access to consumables. Groups brainstormed the design of an mHealth US-guided biopsy training program, preferring a train-the-trainer format combining in-person teaching with independent modules.

**CONCLUSION:**

A multidisciplinary needs assessment of local stakeholders identified a need for and acceptability of an mHealth-based US-guided biopsy training program in Nigeria.

## INTRODUCTION

Breast cancer incidence is rising in Nigeria and is the leading cause of cancer-related death among Nigerian women.^1^ Although diagnosis by ultrasound (US)–guided breast biopsy is the standard of care in high-income countries (HICs), in most low- and middle-income countries (LMICs), including Nigeria, breast cancer is often diagnosed by either palpation-guided biopsy, which is less accurate, or surgical excision, which increases morbidity and cost.^2^ To date, the prohibitive cost of US devices has limited their availability for US-guided biopsy. In this setting, hand-held, battery-operated point-of-care mobile health (mHealth) US devices offer an affordable and sustainable solution.^3,4^

**CONTEXT**

**Key Objective**
Mobile health–based imaging devices have the potential to increase access to ultrasound (US)–guided biopsy; a needs assessment can help identify potential challenges and strategies for implementation. What are Nigerian clinicians’ current practices, training needs, and attitudes regarding US-guided biopsy?
**Knowledge Generated**
Most respondents (n = 54; representing 22 hospitals in Nigeria) reported dissatisfaction with the way breast biopsies are currently performed at their hospital; fewer than one third of radiologists surveyed regularly performed US-guided breast biopsy. Respondents expressed strong interest in creating a US-guided breast biopsy training program focused on radiologists but discussed concerns, including technologic infrastructure and staff workload, which may affect implementation.
**Relevance**
The development of a train-the-trainer US-guided breast biopsy training program is a potential strategy for sustainable capacity building that could positively affect breast cancer control in Nigeria.

Although radiologists in most HICs are trained to perform US-guided breast biopsies during residency and/or fellowship training, no US-guided breast biopsy training program currently exists in Nigeria.^5^ Because of the scarcity of trained radiologists and the urgent need for skilled providers, an accelerated US-guided breast biopsy training program leveraging both remote and on-site training opportunities is needed.

The introduction of US-guided breast biopsy would affect multiple specialists (ie, surgeons, radiologists, pathologists, and nurses) involved in breast cancer diagnosis and treatment. Therefore, when designing a US-guided breast biopsy training program, it is critical to solicit input from all these various stakeholder groups. The objective of this research was to conduct a comprehensive needs assessment among oncology specialists in Nigeria to understand current US use, diagnostic challenges, and training needs to inform the development of an mHealth-based US-guided breast biopsy training program.

## METHODS

### Intervention

The mhealth US intervention consists of a Health Insurance Portability and Accountability Act–compliant application that can be downloaded onto either a tablet or smartphone. The device is then connected by USB to a broadband linear array transducer, a high-frequency US probe with a scan depth of up to 12 cm, which is perfect for breast imaging. Placing the probe on the skin will produce a high-resolution image of the breast on the application. The probe is battery operated and provides 4.5 hours of continuous scan time without a constant energy source.

To perform a breast biopsy, the US probe uses sound waves to create an image of the breast mass, which is displayed via the application. Once the mass for biopsy is identified, a superficial and deep injection of local anesthetic is administered. Once the area is numb, the radiologist makes a small incision and inserts a thin needle to remove samples of tissue or cells.

### Needs Assessment Design and Setting

**FIG 1:**
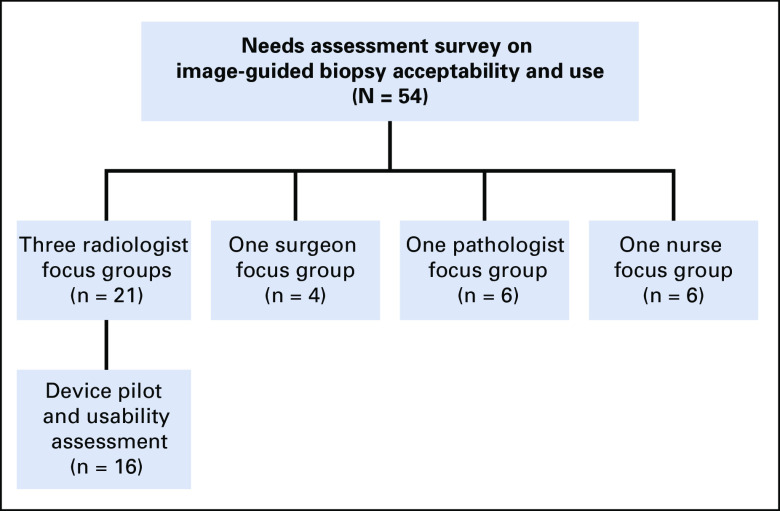
Needs assessment design.

We conducted a needs assessment, approved by the institutional review board, employing both qualitative and quantitative methods. We deployed three assessment metrics: a needs assessment survey, a technology usability survey, and focus group discussions (Fig 1).

Data collection occurred during the 2019 Sixth Annual Symposium of the African Research Group for Oncology (ARGO), a National Cancer Institute–recognized cancer consortium that aims to improve outcomes for patients with cancer in Nigeria. The symposium was hosted by the Obafemi Awolowo University Teaching Hospitals Complex (OAUTHC) in Ile-Ife, Nigeria. This setting presented an opportunity to simultaneously survey diverse stakeholders from across Nigeria.

### Quantitative Assessment

We developed a 27-item needs assessment survey to understand specific barriers, technologic capacity, knowledge, use, and attitudes as regards US-guided breast biopsy and to assess the level of interest in a training program. The survey was developed collaboratively by study team members with local, clinical, and methodologic expertise (K.A.L., T.M.A., A.D.O., E.J.S., and O.I.A.) and included closed and open-ended items organized into three domains: demographics and current practice, workflow and institutional attitudes, and training program interest. Questions in the second domain were adapted from the Organizational Readiness to Change Assessment, a validated scale commonly used to evaluate implementation projects in health services research.^6^ All conference attendees were invited to anonymously participate in this survey, distributed via paper questionnaire.

As the intended participants in the anticipated mHealth US-guided biopsy program, radiologist attendees were invited to take part in a day-long workshop using the device. During this workshop, participants were given an initial demonstration of the US probe and application, connected to a 2018 10.5-inch Samsung Galaxy Tablet (Samsung, Seoul, South Korea). Then, they performed a simulated biopsy on a phantom breast, supervised by experienced trainers (A.D.O. and E.J.S.). After the workshop, radiologists completed an additional technology usability assessment adapted from previously validated measures.^7^ Recognizing that existing computer literacy assessments are not validated in the sub-Saharan African context, a member of our study team (T.M.A.) selected relevant candidate items from prior literature and developed additional items to address technology/infrastructure reliability and access. The new measure was then reviewed and revised for face validity by team members familiar with the local context (O.I.A., A.D.O., T.P.K., and E.J.S.). After a hands-on demonstration of the mHealth device, radiologists completed a paper copy of the anonymous survey.

### Quantitative Analysis

Survey responses were entered into a deidentified database and analyzed descriptively. Frequencies were calculated for categorical data; means, medians, standard deviations, and ranges were calculated for continuous data and Likert scale items. Open-ended responses were aggregated and analyzed in NVivo Pro (version 12.0).^8^

### Qualitative Assessment

We conducted focus groups across radiologists, surgeons, pathologists, and nurses. Given that radiologists would be the primary participants in a US-guided breast biopsy program, we oversampled the radiologist group, stratifying the discussions by three geographic zones (southeast, north, and southwest), to identify key differences in current practice and need. A convenience sample was used for recruitment; after specialty-specific workshop sessions, a member of our study team approached attendees, explained the purpose of the research, and asked if they had interest in or availability for participating in a group discussion. Focus groups were led by an experienced qualitative methods specialist (QMS; K.A.L.) and were conducted according to established methodologic guidelines.^9^ The following discussion topics were developed collaboratively by team members with content and methodologic expertise (K.A.L., E.J.S., and A.D.O.): process of diagnosing and assessing extent of disease, current barriers and challenges to diagnosis, interest in incorporating US-guided breast biopsy, clinical and institutional/workflow implications of US-guided breast biopsy, and training preferences and goals. The number and size of the focus groups were selected to help achieve data saturation, defined in this study as the point at which all themes were fully explored, with no new information arising from additional questioning.^9^ Each group was audio recorded and transcribed for analysis. The facilitator also took notes on key themes and quotes during the discussion and created a summary after each interview.

### Qualitative Analysis

All notes and transcripts were analyzed using inductive thematic content analysis.^9-11^ The QMS and a trained research assistant (J.A.V.) independently coded each transcript, highlighting significant statements within each domain.^11^ Then, the coding team met to reach consensus regarding primary themes for that transcript, producing a summary document with illustrative quotes. In the final phase, the team analyzed summary documents to identify key themes observed across transcripts, making note of any significant divergences between stakeholder groups.

## RESULTS

### Needs Assessment Survey

Needs assessment surveys were completed with a 55% response rate (n = 54 of 98). Demographics are summarized in Table 1. There was a relatively even balance of male (46.3%) and female respondents (53.7%). A majority were radiologists (46.2%) or surgeons (35.2%) practicing for a mean of 10.38 years (standard deviation [SD], 7.77 years).

**TABLE 1:**
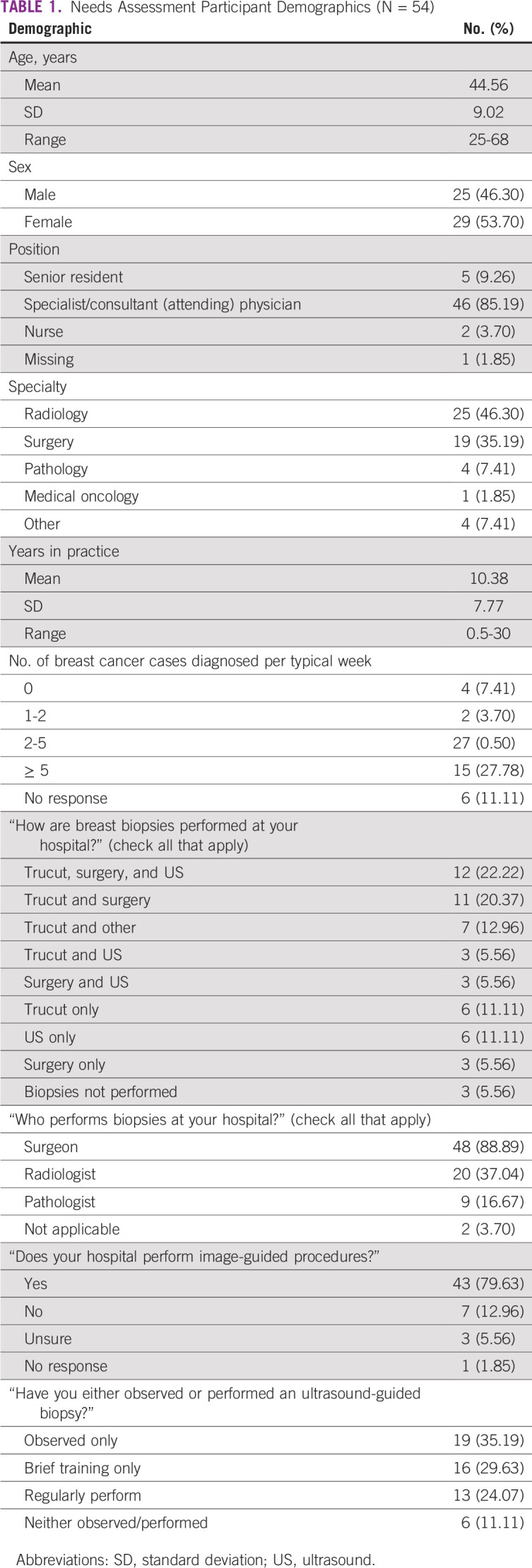
Needs Assessment Participant Demographics (N = 54)

Respondents represented 22 hospitals at the ARGO Symposium, the most common being OAUTHC (n = 8). Half (n = 27 of 54) of the respondents diagnosed two to five cases of breast cancer weekly. In open-ended responses, the most frequently reported challenges to diagnosing cancer included the high cost of biopsies (n = 10), delayed histology and inadequate access to immunohistochemistry (n = 11), faulty and/or lack of imaging equipment (n = 10), inadequate training (n = 3), and late presentation of patients for care (n = 3). Available imaging equipment reported included ultrasound (n = 32), computed tomography (n = 13), mammography (n = 4), magnetic resonance imaging (n = 3), and x-ray (n = 1); 11 respondents indicated that they did not have or were not aware of imaging equipment available at their hospital. The majority (88.9%) responded that surgeons performed biopsies at their hospital, and 37% responded that radiologists performed biopsies; notably, only one respondent indicated that radiologists were solely responsible for performing biopsies at his or her hospital. Most respondents indicated that some combination of trucut, surgical, and US-guided breast biopsies were performed at their hospital; six indicated that only US-guided breast biopsies were performed, whereas another six indicated that only blind (palpation-guided) biopsies were performed. Seven indicated other forms of biopsies aside from surgical, trucut, and US-guided biopsies, including cell block, fine-needle aspiration cytology, and hematoma localized US-guided excision. Only 24% of respondents regularly performed US-guided breast biopsy; 35% had observed the procedure. When broken down by specialty, approximately one third (32%) of radiologists in the sample regularly performed US-guided breast biopsy.

Likert responses illustrating attitudes toward US-guided breast biopsy are listed in Table 2, with a score of 1.0 indicating “strongly disagree” and 5.0 indicating “strongly agree.” Respondents on average disagreed with the statement, “I am satisfied with the way breast biopsies are currently performed at my hospital” (mean, 2.68; SD, 1.19). Overall, respondents held positive attitudes toward incorporating US-guided biopsy into the diagnostic process, showing strong agreement with the statement, “I think US-guided breast biopsies would improve patient time to diagnosis at my hospital” (mean, 4.39; SD, 1.05).

**TABLE 2:**
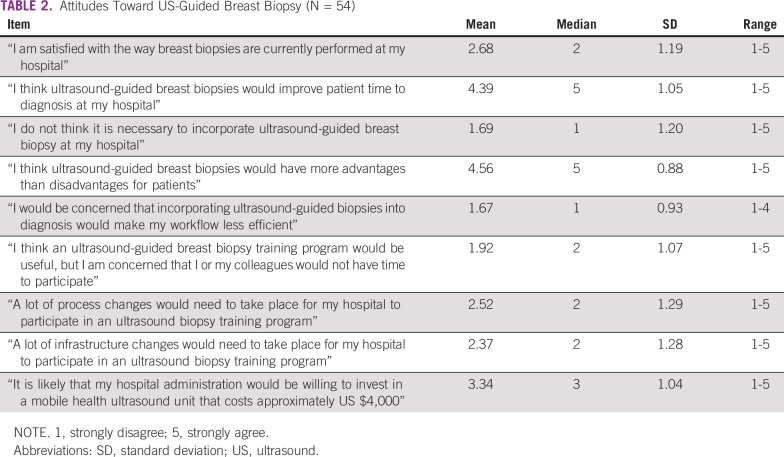
Attitudes Toward US-Guided Breast Biopsy (N = 54)

Respondent preferences and interest in an US-guided biopsy training program are summarized in Table 3. Seventy-eight percent expressed strong interest in creating a US-guided biopsy training program (mean, 4.68; SD, 0.83). A majority indicated that they would be interested in a program that included opportunities for electronic or tablet-based learning (mean, 4.74; SD, 0.48). In open-ended responses, respondents felt that the main benefits of US-guided biopsy included reduced time to diagnosis, increased accuracy of diagnosis, and early detection, leading to improved disease management.

**TABLE 3:**
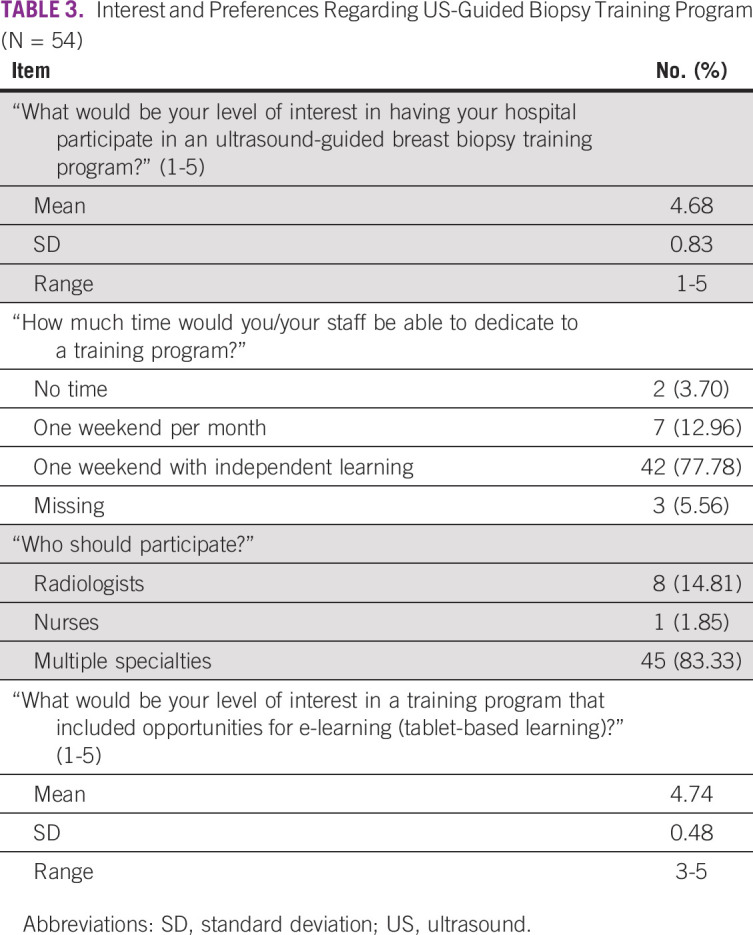
Interest and Preferences Regarding US-Guided Biopsy Training Program (N = 54)

### Comfort With Technology and mHealth Usability Assessment

Radiologist technology surveys (Tables 4 and 5) were completed with a 76% response rate (n = 16 of 21). Overall, respondents self-rated high levels of comfort with and acceptability of using mobile applications in clinical settings. Seventy-five percent responded that their hospital did not use an electronic medical record. Almost all respondents (81.3%) had previously participated in online learning courses. Radiologists also rated the mHealth US device to be usable and acceptable after initial training and stated in open-ended responses that the mHealth device was easy to learn and convenient and had potential to improve health care delivery.

**TABLE 4:**
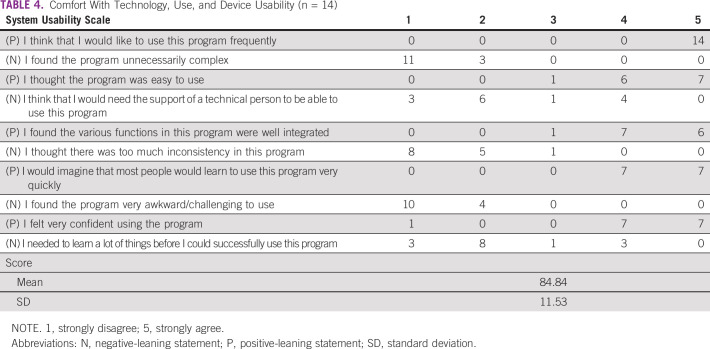
Comfort With Technology, Use, and Device Usability (n = 14)

**TABLE 5:**
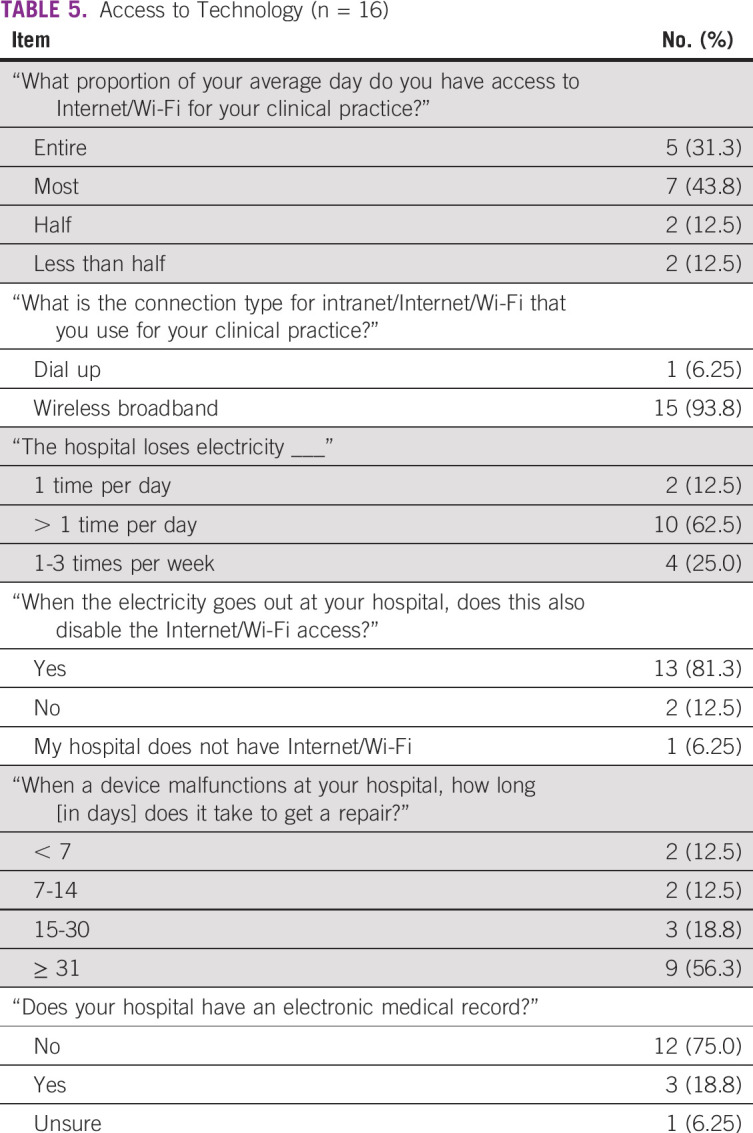
Access to Technology (n = 16)

Respondents reported issues with electricity and Internet access. A majority (62.5%) indicated that hospital electricity supply was lost more than once per day; 81.3% noted that when electricity was interrupted, Internet access was also disabled. Ten responded that it could take > 1 hour for electricity to be restored. More than half (56.3%) indicated that they were responsible for funding their own Internet/Wi-Fi at their clinical practice. Nine responded that it could take > 1 month for a malfunctioning device to be repaired at their hospital.

### Focus Groups

Six focus groups consisting of surgeons (n = 4), radiologists (three groups; n = 21), pathologists (n = 6), and nurses (n = 6) lasted 2045 minutes. We were able to obtain data saturation on three key themes related to current practices and challenges, training preferences and goals, and potential institutional impacts and workflow implications, which are summarized in Table 6.

**TABLE 6:**
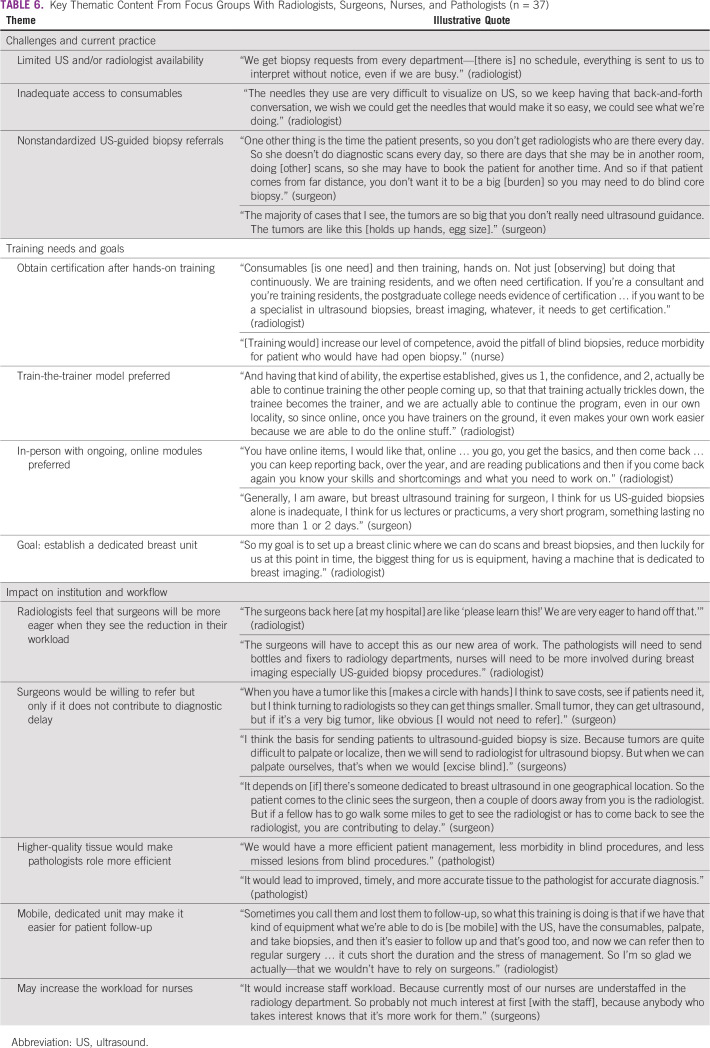
Key Thematic Content From Focus Groups With Radiologists, Surgeons, Nurses, and Pathologists (n = 37)

#### Theme one: current practice and challenges.

Most hospitals had one to two US machines shared between departments, limiting US-guided breast biopsies to certain days of the week and contributing to delays in patient care. Access to consumables was an issue; radiologists noted that the cost of a biopsy needle was approximately one third of the cost of the procedure itself. Limited time with the US machine and procedure costs made it difficult to screen and follow up with patients. Radiologists from northern hospitals noted that their patients often faced greater financial constraints compared with patients from other regions of Nigeria and expressed greater concern about incessant power outages at their practice.

Although most surgeons had the option to refer a patient for US-guided breast biopsy, blind biopsies were frequently performed, largely determined by tumor size. As one surgeon stated, “When the tumor is so big, you don’t really need US guidance.” Surgeons stated that a radiologist’s availability often determined whether a US-guided biopsy was performed; because many patients needed to travel long distances, surgeons were more likely to perform a blind biopsy if the radiologist was unable to do the biopsy the same day.

All pathologists and nurses were staff at OAUTHC. Pathologists reported higher-quality tissue samples since the introduction of US-guided biopsy at their institution, facilitating faster analysis, more accurate diagnosis, and prompt patient care. Nurses working on the inpatient floor affirmed that it could be difficult to transport weak perioperative patients across the hospital campus for imaging follow-up, expressing interest in a transportable US unit. However, without hospital-provided Wi-Fi, nurses were concerned that they would need to use their personal data plans when interacting with the mHealth device.

#### Theme two: training preferences and goals.

There was consensus among focus group participants that radiologists would be the most appropriate specialty to participate in a US-guided breast biopsy training program. Participants had a strong preference for a train-the-trainer model, where the previous cohort mentors the next group of trainees, to facilitate sustainable capacity building within their own hospital. Participants felt that obtaining official certification upon completion of the program would increase institutional support for training. The groups expressed interest in an ongoing training model to foster collaboration between members of the cohort. However, radiologists from northern hospitals felt a single, week-long training program might be more feasible for clinicians coming from areas where travel was difficult. Training goals included obtaining skills needed to establish a dedicated breast unit at their hospital, as well as increased screening and outreach to rural areas.

#### Theme three: institutional and workflow implications.

Across groups, participants articulated the potential impacts of implementing US-guided biopsy. Both surgeons and pathologists anticipated US-guided breast biopsy would shorten time to diagnosis, especially for small tumors. Pathologists felt that the introduction of US-guided breast biopsy would allow them to work with smaller, earlier-stage, and cleaner samples. Surgeons agreed that, if US-guided breast biopsy were available, they would be willing to refer to radiologists “if the tumor is small.” However, surgeons also felt that if a tumor were “very big, like obvious,” they would carry out palpation-guided biopsy to reduce patient costs.

Although surgeons anticipated that incorporating US-guided breast biopsy would reduce their workload, they felt that it might increase the burden on understaffed nurses, who assist with procedures, and radiologists. Similarly, although nurses felt that US-guided biopsy would benefit patients, most felt that supporting US-guided breast biopsy procedures could be burdensome. Many nurses noted that they were paid per month rather than hourly; there was concern that additional US procedures would increase their hours without an associated increase in pay. Although pathologists expressed some concern that an increase in US-guided breast biopsy would cause some workflow constraints (ie, an increase in sample volume with limited staff capacity), they felt this would be mitigated by the higher tissue quality.

## DISCUSSION

Overall, Nigerian clinicians expressed a need for and interest in incorporating US-guided breast biopsy into breast cancer diagnosis. An ongoing train-the-trainer model that offers online modules punctuated by hands-on seminars was preferred. Obtaining certification in this procedure was the primary goal of many potential trainees to establish a dedicated breast radiology unit in their hospital.

Findings from this needs assessment informed an implementation plan for the training program, guided by the Consolidated Framework for Implementation Research^12^ because of its focus on the context (inner and outer settings) of intervention deployment. For instance, although interest in the training was high, especially among radiologists, technology and electricity access emerged as concerns. Radiologists and nurses noted that they often needed to self-fund their Internet access in the clinic, and daily power outages were common. To address this inner setting issue, it will be necessary to provide Wi-Fi in addition to the mHealth devices.

Although there seemed to be consensus that US-guided breast biopsies have clinical benefits, institutional impacts, also characteristics of the inner setting, need to be considered. Reluctance by surgeons to refer patients with large tumors for imaging is a factor that may affect US-guided breast biopsy implementation. At many hospitals, radiologists could only see patients if they received a referral from a surgeon. Therefore, to integrate US-guided breast biopsy, it will be critical for the surgeons to be on board, and this may need to be specifically addressed in educational modules. The establishment of a specialized multidisciplinary breast clinic, identified by focus group participants as a primary goal, may help address this concern as radiologists and surgeons begin to work closely on a team.

Key outer setting concerns raised by providers, including high cost of care, reimbursement structures, and limited provider availability, present challenges to implementation and highlight the importance of training multiple radiologists per institution. Introducing this technology through a train-the-trainer model can be a sustainable to way to address provider availability; staggering training means that many radiologists can be trained without having to leave their institution. This will also ensure that more radiologists per site have the capacity to perform US-guided biopsies, thus reducing the workload for each individual radiologist and decreasing patient wait times. With the expansion in imaging availability, there is the potential that patient tumors can be detected earlier, reducing overall treatment costs.

This study acknowledges the following limitations: relatively small sample size and possible response bias, in that only individuals with strongly positive or negative experiences were willing to share feedback. In sampling symposium attendees, there may have been selection bias toward a population interested in innovation and more open to learning new techniques. However, this study was strengthened by multiple methods of data collection and sampling of clinicians from diverse regional and disciplinary backgrounds.

In conclusion, this needs assessment of local stakeholders identified a clear need for and acceptability of an mHealth-based US-guided breast biopsy training program. Such a program will expand access to technology that will enable Nigerian clinicians to expeditiously make diagnoses, allowing the entire system of breast cancer early detection and diagnosis to move forward as efforts are made to downstage disease at diagnosis. The development of an mHealth-based US-guided breast biopsy training program is a strategy for sustainable capacity building that could positively affect breast cancer control in Nigeria.

## APPENDIX

Members of the African Research Group for Oncology Collaborative: Krishna Patel, MD; Adedeji Egberongbe, MBBS; Nneka Iloanusi, MBBS; Adenike Adeniji-Sofoluwe, MBBS; Olubukola Omidiji, MBBS; Faosat Jinadu, MBBS; Adeyanju Akinola, MBBS; Millicent Obajimi, MBBS; Zainab Mustapha, MBBS; Yolanda Bryce, MD; Olalekan Olasehinde, MBBS; Victoria Mango, MD; Anya Romanoff, MD; Oluwole Odujoko, MBBS; Marcia Edelweiss, MD; Peter Ntiamoah, PhD; Olugbemiga O. Ayoola, MBBS; Lauren A. Koranteng, PharmD; and Margaret Barton-Burke, PhD, RN.
